# Heat-Killed *Lacticaseibacillus paracasei* Ameliorated UVB-Induced Oxidative Damage and Photoaging and Its Underlying Mechanisms

**DOI:** 10.3390/antiox11101875

**Published:** 2022-09-21

**Authors:** Jing Xu, Xiaofang Zhang, Yan Song, Bin Zheng, Zhengshun Wen, Miao Gong, Lingting Meng

**Affiliations:** Food and Pharmacy College, Zhejiang Ocean University, Zhoushan 316022, China

**Keywords:** heat-killed *Lacticaseibacillus paracasei*, skin cells, oxidative damage, photoaging, UVB

## Abstract

Ultraviolet B (UVB) radiation is a major environmental causative factor of skin oxidative damage and photoaging. *Lacticaseibacillus paracasei* is a well-known probiotic strain that can regulate skin health. The present study investigated the effects of heat-killed *Lacticaseibacillus paracasei* (PL) on UVB linked oxidative damage and photoaging in skin cells (Normal human dermal fibroblast (NHDF) cells and B16F10 murine melanoma cells). Results demonstrated that: (1) PL prevented UVB-induced cytotoxicity relating to decreased DNA damage in NHDF and B16F10 cells; (2) PL alleviated UVB-induced oxidative damage through increasing GSH content, as well as antioxidant enzyme activities and mRNA levels (except MnSOD activity and mRNA levels as well as CAT mRNA level) relating to the activation of Sirt1/PGC-1α/Nrf2 signaling in NHDF cells; (3) PL attenuated UVB-induced photoaging was noticed with a decrease in the percentage of SA-β-gal positive cells in NHDF cells model. Moreover, PL attenuated UVB-induced photoaging through exerting an anti-wrinkling effect by enhancing the type I collagen level relating to the inhibition (JNK, p38)/(c-Fos, c-Jun) of signaling in NHDF cells, and exerting an anti-melanogenic effect by suppressing tyrosinase and TYRP-1 activity and/or expressions relating to the inhibition of PKA/CREB/MITF signaling in B16F10 cells. In conclusion, PL could ameliorate UVB-induced oxidative damage and photoaging. Therefore, PL may be a potential antioxidant and anti-photoaging active ingredient for the cosmetic industry.

## 1. Introduction

Skin is the first barrier that protects human body from environmental hazards [1]. Ultraviolet (UV) irradiations (including UVA, UVB and UVC) are major exogenous stimulators that result in accumulative skin injury [2]. Typically, UVB is the most threatening spectrum for skin health [3,4]. Prolonged exposure to UVB could cause skin oxidative damage and photoaging [5,6]. In recent years, some natural extracts and artificial compounds, such as xanthophyll, polyphenol, retinyl palmitate and hydroquinone, had been used as active ingredients in antioxidant and anti-photoaging products [7,8,9,10]. However, these above ingredients are costly, and even have some side effects. Therefore, it is of great significance to find inexpensive and safe sunscreen ingredients to ameliorate UVB-induced skin oxidative damage and photoaging. Probiotics are live bacteria that can benefit the host’s health [11]. Several studies reported that oral live probiotics exerts the effects beyond the gut and confers benefits at the skin level, such as reinforcing skin barrier function, decreasing skin sensitivity and relieving skin inflammation [12,13,14]. However, the safety profile with the use of live probiotics is still a matter of debate [15]. The main risks for oral probiotics are systemic infections due to translocation, particularly in a population with weak immunity [16]. To avoid these risks, there is an increasing interest in replacing live probiotics with non-viable microorganisms or microbial cell extracts, mainly probiotics inactivated by heat treatment (termed heat-killed probiotics), due to their long shelf life, safety and beneficial effects for skin health [15]. Recent research studies have found that oral administration of heat-killed probiotics could safely improve skin health at a low cost, by reducing transepidermal water loss and enhancing skin elasticity [17,18]. Nevertheless, there is a scarcity of research on the influence of heat-killed probiotics on skin oxidative stress and photoaging. *Lacticaseibacillus paracasei* is a well-established probiotic isolated from the intestines of healthy adults [19]. To date, only two reports are available on the effects of inactivated *Lacticaseibacillus paracasei* by heat treatment, heat-killed *Lacticaseibacillus paracasei* (PL), on the skin health. Indeed, Moroi et al. (2011) found that a supplementary diet containing PL may have some benefits as a complementary therapy for adult atopic dermatitis patients who are managed with the conventional treatment [20]. Tsai et al. (2021) reported that PL could promote healing in the experimental cutaneous wounds of a mouse [21]. However, there are no studies regarding the effect of PL on skin oxidative stress and photoaging. Previous studies found that heat treatment of *Lacticaseibacillus paracasei* could produce thalli ingredients (such as lipoteichoic acid, peptidoglycan, exopolysaccharides and cell surface proteins) and metabolites (such as probiotics peptides, organic acids, flavonoid compounds, alcohols and polyphenolic compounds) [22,23]. It was reported that lipoteichoic acid isolated from probiotics could alleviate oxidative damage and photoaging in human dermal fibroblast cells [24,25,26]. These observations indicated that PL may ameliorate UVB-induced skin oxidative damage and photoaging, which is worthy of further study.

Skin oxidative damage could be induced by the increase in reactive oxygen species (ROS) generation upon repeated exposure to UVB irradiation [5]. A high level of ROS interacts with both lipid peroxidation and protein oxidation, whose biomarkers are malondialdehyde (MDA) and protein carbonyl (PC), respectively [27]. In general, the protective effect against skin oxidative damage was related to nonenzymatic compounds (glutathione(GSH)) and antioxidant enzymes (including superoxide dismutase (SOD), catalase (CAT), glutathione peroxidase (GPx), glutathione S-transferases (GST) and glutathione reductase (GR)) [28]. Antioxidant enzyme activities are partly dependent on antioxidant enzyme gene transcription, which could be regulated by NF-E2-related factor 2 (Nrf2) signaling in skin [27]. Moreover, Lee et al. (2022) revealed that Nrf2 could be activated by silent information regulator T1 (Sirt1)/peroxisome proliferator-activated-recptor-γ coactivator-1α (PGC-1α) signaling pathway in human dermal fibroblasts [29]. However, there is no report concerning the effect of PL on skin oxidative damage and underlying molecular mechanisms. It was reported that heat-killed *Lactobacillus* could decrease the MDA level in the serum of mice [30]. Heat-killed *ruminococcus albus* protected neurons from oxidative damage by reducing ROS levels and increasing SOD and GSH levels in rats [31]. Moreover, Luo et al. (2022) found that *Lactobacillus rhamnosus* peptides alleviated oxidative damage by enhancing Nrf2 expression in human colonic epithelial cells [32]. Flavonoids could enhance Sirt1 and PGC-1α protein expressions in human HepG2 cells and the skeletal muscle L6 cells in mice, respectively [33,34]. These findings suggest that PL may ameliorate UVB-induced skin oxidative damage relating to Sirt1/PGC-1α/Nrf2 signaling pathway, which is worthy of further study.

Skin photoaging is usually characterized by wrinkle formation and hyperpigmentation [5]. Type I collagen breakdown is a major cause of skin wrinkle formation [25]. Type I procollagen (COL1A1) is a precursor of type I collagen and can be degraded by matrix metalloproteins (MMPs) [35]. It was reported that MMPs levels could be regulated by mitogen-activated protein kinase (MAPK, including ERK, JNK, and p38)/phosphorylating activator protein-1 (AP-1, including c-Jun and c-Fos) signaling pathways in skin photoaging process [25]. Moreover, skin hyperpigmentation disorder can result from the overproduction of melanin induced by UVB irradiation [5]. Melanogenesis is a physiological melanin-producing process and occurs through enzyme-catalyzed reactions involved in melanogenic enzymes, such as tyrosinase (TYR), tyrosinase-related protein 1 (TYRP-1) and tyrosinase-related protein 2 (TYRP-2) [36]. Microphthalmia-associated transcription factor (MITF) is the master transcription factor in melanogenesis, initiating the transcription of genes encoding TYR, TYRP-1 and TYRP-2 [37]. MITF could be activated by phosphorylated cAMP-response element-binding protein (p-CREB) via the activation of cyclic AMP/protein kinase A (cAMP/PKA) signaling pathway in skin [36]. However, no study has focused on the effect of PL on skin photoaging and the possible mechanism. Lee et al. (2021) found that heat-killed *Bifidobacterium bifidum* could reduce the secretion level of IL-6 in RAW 264.7 macrophage cells [38]. It was reported that the decrease in IL-6 secretion could inhibit type I collagen breakdown through suppressing MMP-1 protein expression in human fibroblast [35]. Kim et al. (2018) revealed that thioredoxin-interacting protein-derived peptide inhibited p38 activation and decreased p-c-Jun protein expression in RAW 264.7 macrophage cells [39]. Moreover, it was reported that tyndallized *Lactobacillus acidophilus* could decrease TYR, TYRP-1 and TYRP-2 gene expressions as well as MITF protein expression relating to inhibit PKA/CREB signaling pathway [37]. These findings suggest that PL may ameliorate skin photoaging through anti-wrinkling and anti-melanogeneic effects relating to MAPK/AP-1 and PKA/CREB/MITF signaling pathways, respectively, which is worthy of further investigation. 

In this study, we hypothesized that PL could reverse UVB-induced skin oxidative damage and photoaging. To confirm this hypothesis, we investigated the effects of PL on antioxidant enzyme activities, type I collagen level and melanin production as well as the potential regulatory mechanisms using normal human dermal fibroblasts (NHDF) and B16F10 murine melanoma cells exposed to UVB irradiation, which may provide a theoretical basis for the development of PL as an active ingredient in skin antioxidant and anti-photoaging products.

## 2. Materials and Methods

### 2.1. PL Preparation

The preparation of PL was performed as described by Shukla et al. (2020) and Arai et al. (2018) [40,41]. *Lacticaseibacillus paracasei* was cultivated and maintained in MRS broth at 37 °C for 24 h. Overnight-cultivated strains were centrifuged at 1200× *g* for 15 min, washed twice with phosphate-buffered saline (PBS), and then washed twice with sterile distilled water. Subsequently, the organisms were suspended in distilled water and heat-killed at 80 °C for 20 min. Effectiveness of heat killing was confirmed by plating on MRS agar and incubated for 72 h at 37 °C. The heated suspension was lyophilized to obtain the dried powder of PL.

### 2.2. UVB Irradiation

UV irradiation was performed using a UVB lamp (JY-T58; wavelength range: 290–320 nm, peak: 312 nm; Zhongshan OURI Optoelectronic Technology, Zhongshan, China). The UVB irradiation intensity was measured using a Waldmann UV meter (model 585100, Waldmann, Villingen-Schwenningen, Germany). The UV irradiation dose for NHDF and B16F10 cells used in this study (30 mJ/cm^2^) is calculated according to the following formula:

UV irradiation dose (mJ/cm^2^) = UVB irradiation intensity (mW/cm^2^) × irradiation time (s).

### 2.3. Cell Culture

NHDF and B16F10 cells were cultured in DMEM supplemented with 10% fetal bovine serum and 1% penicillin-streptomycin, and it was kept incubated at 37 °C in a 5% CO_2_ environment.

### 2.4. MTT Assay

Cell viability was evaluated by MTT assay as described by Ding et al. (2020) [42]. Briefly, NHDF and B16F10 cells were treated with PL (1.0 mg/mL) for 1 h. The treatment concentration of PL here was chosen according to the results of a preliminary experiment (data not shown), in which we treated cells with different concentration gradients of PL (0.0, 0.5, 1.0, 1.5, 2.0 mg/mL). Then, the cells were irradiated with UVB (30 mJ/cm^2^). After 24 h incubation, 100 µL of MTT (2 mg/mL) was added to the cells and incubated for 3 h. Then, 300 μL DMSO was added to the cells, and the absorbance was measured at 540 nm with a microplate reader (Thermo, San Francisco, CA, USA).

### 2.5. TUNEL Assay

DNA damage was assessed by TUNEL assay as described by Xu et al. (2017) [43]. Briefly, NHDF and B16F10 cells were exposed to UVB (30 mJ/cm^2^) and then treated with PL (1.0 mg/mL) for 24 h. The treatment concentration of PL here was identical with that found in the MTT Assay. Thereafter, NHDF and B16F10 cells were washed with PBS and fixed with 4% paraformaldehyde for 25 min, washed twice with PBS and then incubated with 0.2% Triton X-100 for 5 min. Subsequently, the cells were incubated with TUNEL reaction mixture for 1 h at 37 °C in the dark, and then stained with DAPI (5 μg/mL) solution for 10 min at room temperature. After being washed three times with PBS, the cells were visualized using a fluorescence microscope (NiKon, Tokyo, Japan).

### 2.6. DCFH-DA Staining Assay

ROS production was determined by DCFH-DA staining assay according to Hu et al. (2019) [44]. Briefly, NHDF cells were irradiated with UVB (30 mJ/cm^2^) before being treated with PL (1.0 mg/mL) for 24 h. The treatment concentration of PL here was chosen according to the results of the preliminary experiment (data not shown), in which the cells were irradiated with UVB (30 mJ/cm^2^) and then treated with different concentration gradients of PL (0.0, 0.5, 1.0, 1.5, 2.0 mg/mL). After being washed two times with PBS, DCFH-DA (10 μM) was added to the cells and incubated at 37 °C for 20 min. Subsequently, the cells were observed under a fluorescence microscope, and the fluorescence intensity of DCFH-DA was measured using a multifunctional microplate reader with the excitation and emission wavelengths of 485 nm and 535 nm, respectively.

### 2.7. Oxidative Damage and Antioxidant Assay

NHDF cells were irradiated with UVB (30 mJ/cm^2^) before being treated with PL (1.0 mg/mL) for 24 h. The method for choosing appropriate PL treatment concentration (1.0 mg/mL) was the same as that found in the DCFH-DA Staining Assay. Then, the cells were collected and lysed on ice using an ultrasonic disintegrator, and the protein concentration was determined according to the Bradford method. MDA, PC and GSH contents as well as the activities of antioxidant enzymes (including Cu/ZnSOD, MnSOD, CAT, GPx, GST and GR) in NHDF cells were measured according to the manufacturer’s instructions (Nanjing Jiancheng Co., Nanjing, China).

### 2.8. β-Gal Assay

Senescence-associated-β-galactosidase (SA-β-gal) was usually used as a marker of cellular senescence and can be measured using senescent cells histochemical staining kit (Sigma Aldrich, St. Louis, MO, USA). Briefly, NHDF cells were exposed to UVB (30 mJ/cm^2^) and then treated with PL (1.0 mg/mL) for 24 h. The method for choosing appropriate PL treatment concentration (1.0 mg/mL) was the same as that found in the DCFH-DA Staining Assay. The cells were fixed in 4% paraformaldehyde and incubated overnight at 37 °C with fresh SA-β-gal stain solution according to the manufacturer’s instruction. The SA-β-gal activity was assessed by using the method originally described by Permatasari et al. [45]. All of the blue-stained cells found in ten fields (5 × 10^5^ cells) were counted under a microscope with ×100 magnification and expressed as the percentage of positive cells.

### 2.9. ELISA Assay

According to the method of Hong et al. (2015) [25], type I collagen level in NHDF cells was measured using enzyme-linked immunosorbent assays (ELISA) assay. NHDF cells were treated with PL (1.0 mg/mL) for 24 h after being irradiated with UVB (30 mJ/cm^2^). The method for choosing appropriate PL treatment concentration (1.0 mg/mL) was the same as that found in the DCFH-DA Staining Assay. Then, the cell medium was collected and harvested to determine type I collagen level according to the manufacturer’s instruction (Nanjing Jiancheng Co., Nanjing, China)

### 2.10. Melanin Generation and Tyrosinase Activity Assays

Melanin generation and tyrosinase activity assays were evaluated by the method of Liu et al. (2021) [46]. B16F10 cells were exposed to UVB (30 mJ/cm^2^) and then treated with PL (1.0 mg/mL) for 24 h. The method for choosing appropriate PL treatment concentration (1.0 mg/mL) was the same as that found in the DCFH-DA Staining Assay. Then, the cells were collected with trypsin and washed twice with PBS, and lysed by 170 μL NaOH solution (1 mol/L) for 1 h at 90 °C. The total amount of melanin per lysate was determined at 405 nm using a microplate reader (Thermo, San Francisco, CA, USA). Moreover, for the tyrosinase activity assay, after 24 h incubation with treatments, the cells were collected and lysed in RIPA lysis buffer. Supernatant fractions were obtained by centrifugation (10,000× *g*, 15 min, 4 °C). Then, cell homogenates containing 80 μg of protein in each and 80 μL L-DOPA (5 mM) were added to the wells in 96-well microplates. The mixtures were incubated at 37 °C for 30 min. After incubation, dopachrome formation was detected by measuring absorbance at 492 nm using a microplate reader.

### 2.11. qRT-PCR Assay

qRT-PCR Assay was performed according to the method of Song et al. (2021) [47]. Briefly, NHDF and B16F10 cells were exposed to UVB (30 mJ/cm^2^) and then treated with PL (1.0 mg/mL) for 24 h. The treatment concentration of PL here was identical with that found in the above experiments. Total RNA was isolated using RNAiso Plus Kit (Takara, Kusatsu, Japan) according to the manufacturer’s instructions followed by DNase I treatment. The quality and quantity of the total RNA were assessed by agarose gel electrophoresis at 1% and by spectrophotometric analysis at 260 and 280 nm. Subsequently, RNA was reversed transcribed into cDNA with PrimeScriptTM RT Reagent Kit (Takara, Dalian, China). Then, real-time PCR reactions were performed on a CFX96™ Real-Time PCR Detection System (Bio-Rad, Laboratories, Inc., Hercules, CA, USA) using the PrimeScript RT Reagent kit (Takara Bio, Inc., Kusatsu, Japan). The primers sequences of genes are shown in Table S1. β-actin were used as the reference gene according to the data of preliminary tests (not shown here). Amplification was performed in a final volume of 15 μL containing 2 μL cDNA template. After the amplification phase, a melt curve analysis was performed to confirm the specificity of the amplification reaction. The primer amplification efficiencies of these genes were approximately 100%. The relative mRNA level of each treatment was calculated using 2^−∆∆CT^ method.

### 2.12. Western Blotting Assay

NHDF and B16F10 cells treatment with UVB (30 mJ/cm^2^) and PL (1.0 mg/mL) were identical with those in qRT-PCR assay. The procedure of western blotting assay was performed as described by Song et al. (2020) [48]. Briefly, protein concentrations were determined by a BCA assay kit (Beyotime Biotechnology Inc., Shanghai, China). An equal amount of protein sample was separated by SDS-PAGE, and then transferred to polyvinylidene difluoride (PVDF) membranes (Millipore, Billerica, R0BB27122, MA, USA). After transfer, the membranes were blocked with 5% nonfat milk at room temperature for 2 h, and then incubated overnight at 4 °C with the corresponding primary antibody (Sirt1, PGC-1α, Nrf2, Keap1(Kelch-like ECH-associated protein 1), p-ERK, ERK, p-JNK, JNK, p-p38, p38, COL1A1, p-c-Fos, c-Fos, p-c-Jun, c-Jun, TYR, p-PKA, PKA, p-CREB, CREB, MITF, Lamin B1 and β-actin antibodies) before being incubated with horseradish peroxidase (HRP)-conjugated secondary antibody for 2 h. The protein bands were detected by chemiluminescence with an imaging system, and then analyzed using Image J software. The expressions of target proteins were showed relative to those observed in control groups. 

### 2.13. Statistical Analyses

In this study, the results were showed as mean ± standard deviation (SD). All data were subjected to one-way analysis of variance (ANOVA), and Duncan method was used to test significant differences among trial groups using SPSS 23.0 (SPSS Inc., Chicago, IL, USA). Notably, *p* < 0.05 was considered statistically significant. Correlations were obtained using Pearson correlation coefficients at the same level of significance. Violin plots were performed using Hiplot platform (https://hiplot.com.cn/, accessed on 20 June 2022).

## 3. Results

### 3.1. The Effects of PL on the Viability and DNA Damage of NHDF and B16F10 Cells

**Figure 1:**
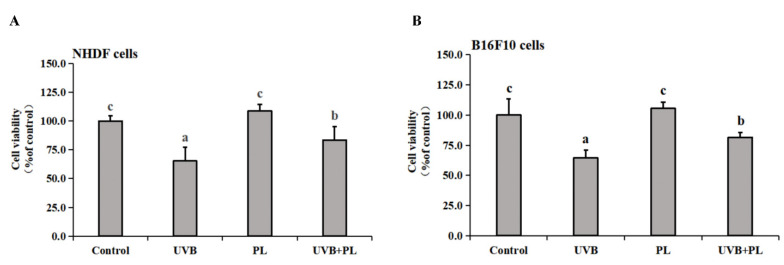
Effects of PL on the viability of NHDF cells (**A**) and B16F10 cells (**B**). Results were expressed as the mean ± SD (*n* = 4). Differences among the variables were assessed using Duncan’s multiple range tests. Values having different letters are significantly different (*p* < 0.05).

The effects of PL on the viability of NHDF and B16F10 are provided in Figure 1. As shown in Figure 1A,B, compared with the control groups, UVB irradiation alone remarkably decreased the viability of NHDF and B16F10 cells (*p* < 0.05), and PL treatment alone had no significant impact on the viability of the above cells (*p* > 0.05). Moreover, compared with the UVB irradiation alone groups, the viability of NHDF and B16F10 cells in UVB+PL groups noticeably increased (*p* < 0.05).

### 3.2. The Effects of PL on DNA Damage in NHDF and B16F10 Cells

**Figure 2:**
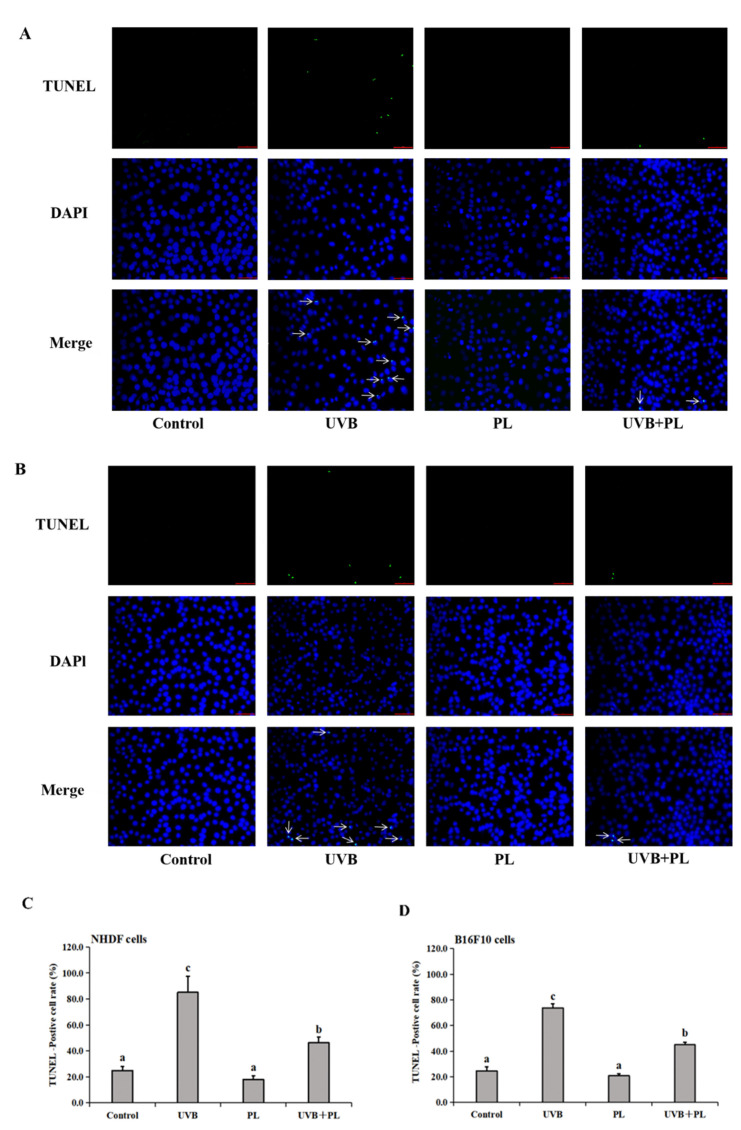
Effects of PL on DNA damage by TUNEL assay in NHDF cells (**A**) and B16F10 cells (**B**). White arrows mark green fluorescence. Quantitative analysis of TUNEL-positive cell rate in NHDF cells (**C**) and B16F10 cells (**D**). Results are expressed as the mean ± SD (*n* = 3). Differences among the variables were assessed using Duncan’s multiple range tests. Values having different letters are significantly different (*p* < 0.05).

The effects of PL on DNA damage in NHDF and B16F10 cells are provided in Figure 2. As shown in Figure 2, compared with the control groups, UVB irradiation alone remarkably increased DNA damage noticed by increased TUNEL-positive cell rate in NHDF and B16F10 cells (*p* < 0.05), and PL treatment alone had no significant effect on DNA damage noticed by the unchanged TUNEL-positive cell rate in the above cells (*p* > 0.05). Moreover, compared with the UVB irradiation alone groups, DNA damage of NHDF and B16F10 cells in UVB+PL groups noticeably decreased, and was evident due to the reduced TUNEL-positive cell rate (*p* < 0.05).

### 3.3. The Effects of PL on Oxidative Damage and Antioxidant Capacity-Related Parameters in NHDF Cells

**Figure 3:**
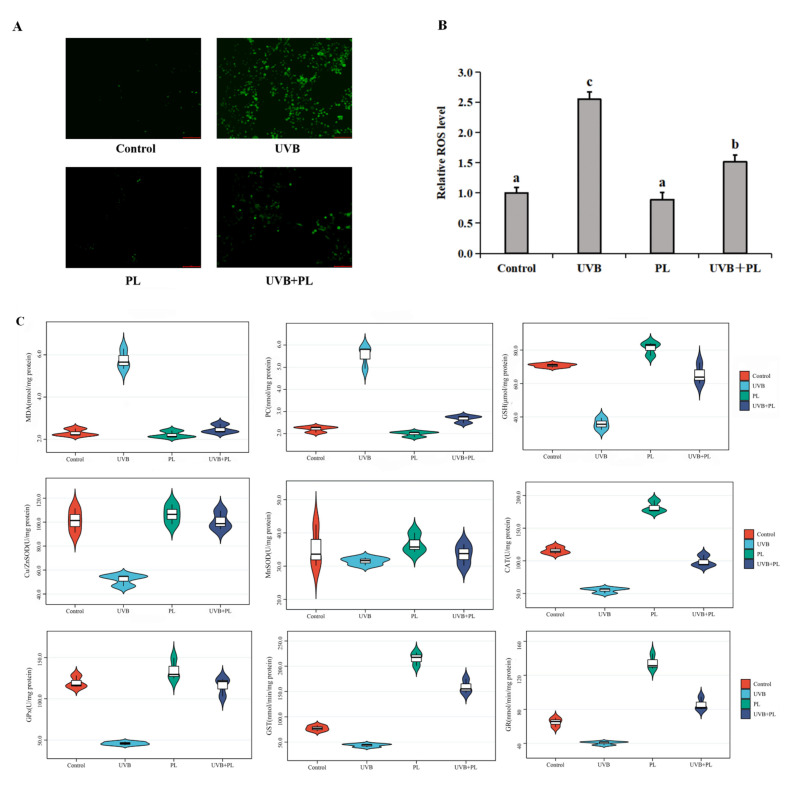

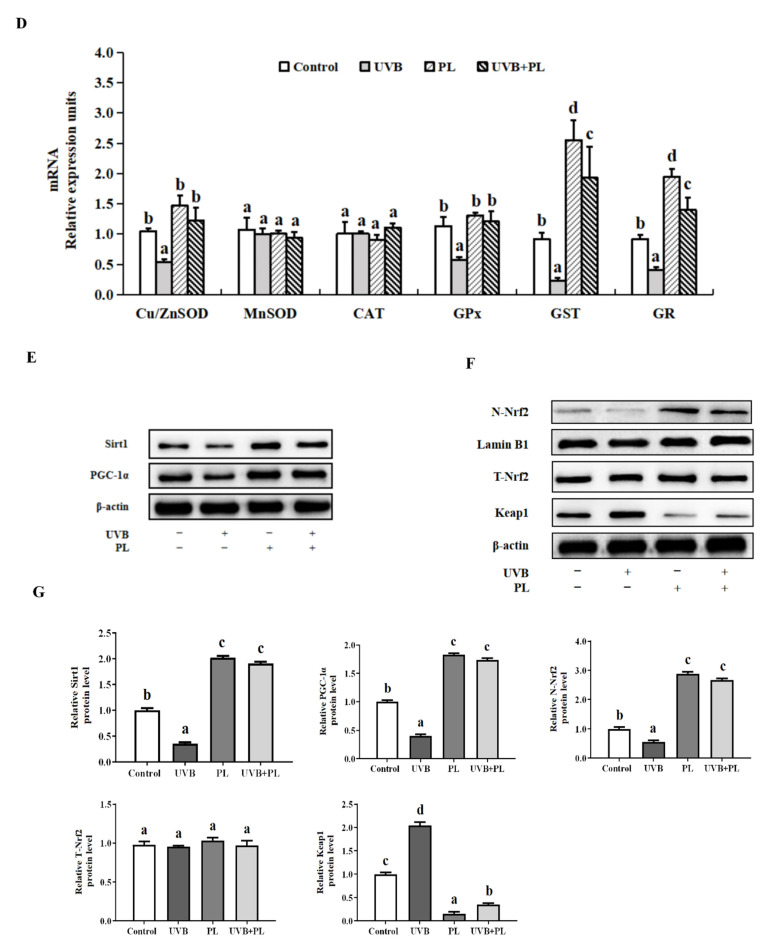
Effects of PL oxidative damage and antioxidant ability in NHDF cells. (**A**,**B**) The relative ROS level, photomicrographs (100 μm) and bar diagram in NHDF cells. (**C**) MDA and PC contents as well as antioxidant enzymes activity; (**D**) The relative mRNA levels of antioxidant enzymes. (**E**–**G**) The relative protein levels of Sirt1, PGC-1α, Nrf2, Keap1. Results are expressed as the mean ± SD (*n* = 3). Differences among the variables were assessed using Duncan’s multiple range tests. Values having different letters are significantly different (*p* < 0.05).

The effects of PL on oxidative damage and antioxidant capacity-related parameters in NHDF cells are provided in Figure 3. As shown in Figure 3A,B, compared with the control group, UVB irradiation alone remarkably increased the relative ROS level (*p* < 0.05), and PL treatment alone had no significant effect on the relative ROS level in NHDF cells (*p* > 0.05). Moreover, compared with the UVB irradiation alone group, the relative ROS level in the UVB+PL group noticeably decreased in NHDF cells (*p* < 0.05).

As shown in Figure 3C, compared with the control groups, UVB irradiation noticeably increased MDA and PC contents (*p* < 0.05), and PL treatment alone had no significant effect on MDA and PC contents in NHDF cells (*p* > 0.05). Compared with the UVB irradiation alone groups, MDA and PC contents in the UVB+PL group noticeably decreased in NHDF cells (*p* < 0.05). Moreover, compared with the control groups, UVB irradiation remarkably or noticeably decreased GSH content as well as Cu/Zn-SOD, CAT, GPx, GST and GR activities (*p* < 0.05), and PL treatment alone had no significant effect on Cu/Zn-SOD and GPx activities (*p* > 0.05) as well as remarkably or noticeably increased GSH content and CAT, GST and GR activities in NHDF cells (*p* < 0.05). Compared with the UVB irradiation alone groups, GSH content as well as Cu/Zn-SOD, CAT, GPx, GST and GR activities in UVB+PL groups remarkably or noticeably increased in NHDF cells (*p* < 0.05). There was no significant difference in MnSOD activity among any of the groups (*p* > 0.05).

As shown in Figure 3D, compared with the control groups, UVB irradiation noticeably decreased Cu/Zn-SOD, GPx, GST and GR mRNA levels (*p* < 0.05), and PL treatment alone had no significant effect on Cu/Zn-SOD and GPx mRNA levels (*p* > 0.05) as well as remarkably increased GST and GR mRNA levels in NHDF cells (*p* < 0.05). Compared with the UVB irradiation alone groups, Cu/Zn-SOD, GPx, GST and GR mRNA levels in the UVB+PL groups noticeably or remarkably increased in NHDF cells (*p* < 0.05). There was no significant difference in MnSOD and CAT mRNA levels among any of the groups (*p* > 0.05).

As shown in Figure 3E–G, compared with the control groups, UVB irradiation noticeably decreased Sirt1, PGC-1α and N-Nrf2 protein levels (*p* < 0.05) as well as noticeably increased Keap1 protein level (*p* < 0.05), and PL treatment alone noticeably increased Sirt1, PGC-1α and N-Nrf2 protein levels as well as remarkably decreased Keap1 protein level in NHDF cells (*p* < 0.05). Compared with the UVB irradiation alone groups, Sirt1, PGC-1α and N-Nrf2 protein levels in UVB+PL groups remarkably increased, as well as the Keap1 protein level in the UVB+PL group, which remarkably decreased in NHDF cells (*p* < 0.05). There was no significant difference in the T-Nrf2 protein level among any of the groups (*p* > 0.05).

### 3.4. The Effects of PL on the Percentage of SA-β-gal Positive NHDF Cells

**Figure 4:**
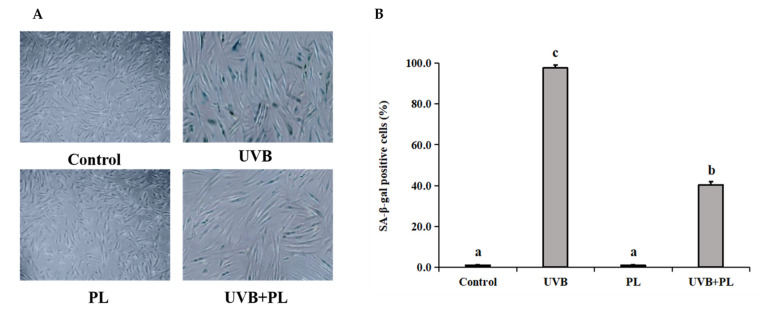
Effects of PL on the percentage of SA-β-gal positive NHDF cells. (**A**,**B**) the percentage of SA-β-gal positive NHDF cells, photomicrographs (×100) and bar diagram in NHDF cells. Results are expressed as the mean ± SD (*n* = 3). Differences among the variables were assessed using Duncan’s multiple range tests. Values having different letters are significantly different (*p* < 0.05).

The effects of PL on the percentage of SA-β-gal positive NHDF cells are provided in Figure 4. As shown in Figure 4A,B, compared with the control group, UVB irradiation alone remarkably increased the percentage of SA-β-gal positive NHDF cells *(p* < 0.05), and PL treatment alone had no significant impact on this index (*p* > 0.05). Moreover, compared with the UVB irradiation alone group, the percentage of SA-β-gal positive NHDF cells in UVB+PL group noticeably decreased (*p* < 0.05).

### 3.5. The Effects of PL on Wrinkle Formation-Related Parameters in NHDF Cells

**Figure 5:**
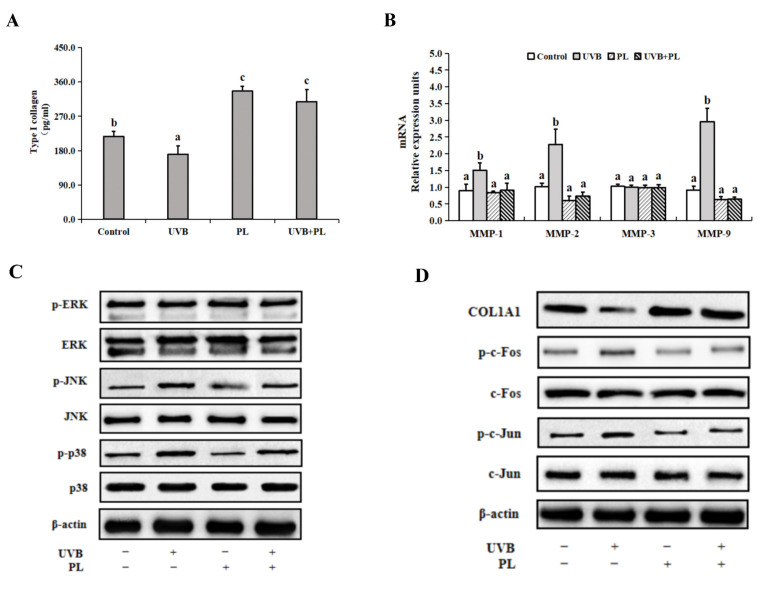

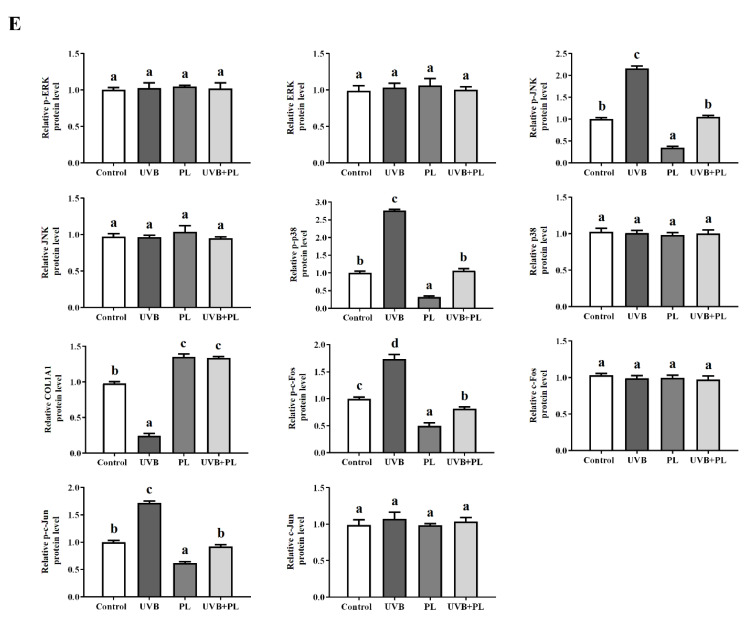
Effects of PL on wrinkles formation-related parameters in NHDF cells: (**A**) Type I collagen content; (**B**) The relative mRNA levels of MMPs; (**C**–**E**) The relative protein levels of p-MAPKs, MAPKs, COL1A1, p-AP-1s, AP-1s; Results are expressed as the mean ± SD (*n* = 3). Differences among the variables were assessed using Duncan’s multiple range tests. Values having different letters are significantly different (*p* < 0.05).

The effects of PL on wrinkle formation-related parameters in NHDF cells are provided in Figure 5. As shown in Figure 5A,B, compared with the control groups, UVB irradiation noticeably decreased type I collagen content as well as noticeably increased MMP-1, MMP-2 and MMP-9 mRNA levels (*p* < 0.05), and PL treatment alone noticeably increased type I collagen content (*p* < 0.05) and had no significant effect on MMP-1, MMP-2 and MMP-9 mRNA levels in NHDF cells (*p* > 0.05). Moreover, compared with the UVB irradiation alone groups, type I collagen content in UVB+PL group remarkably increased, and MMP-1, MMP-2 and MMP-9 mRNA levels in UVB+PL groups noticeably decreased in NHDF cells (*p* < 0.05). There was no significant difference in MMP-3 mRNA level among any of the groups (*p* > 0.05).

As shown in Figure 5C–E, compared with the control groups, UVB irradiation noticeably increased p-JNK, p-p38, p-c-Fos and p-c-Jun protein levels as well as noticeably decreased COL1A1 protein level (*p* < 0.05), and PL treatment alone remarkably or noticeably decreased p-JNK, p-p38, p-c-Fos and p-c-Jun protein levels as well as noticeably increased COL1A1 protein level in NHDF cells (*p* < 0.05). Moreover, compared with the UVB irradiation alone groups, p-JNK, p-p38, p-c-Fos and p-c-Jun protein levels in UVB+PL groups remarkably or noticeably decreased, and the COL1A1 protein level in UVB+PL group remarkably increased in NHDF cells (*p* < 0.05). There was no significant difference in ERK, p-ERK, JNK, p38, c-Fos and c-Jun protein levels among any of the groups (*p* > 0.05).

### 3.6. The Effects of PL on Melanogenesis-Related Parameters in B16F10 Cells

**Figure 6:**
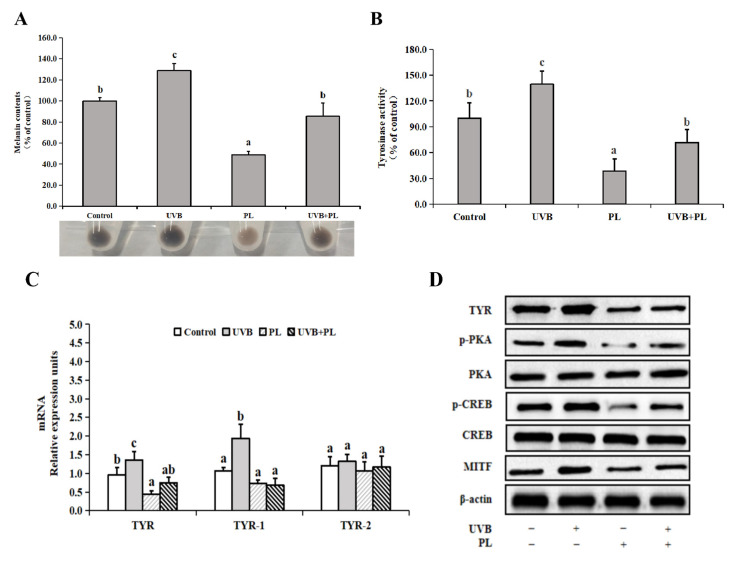

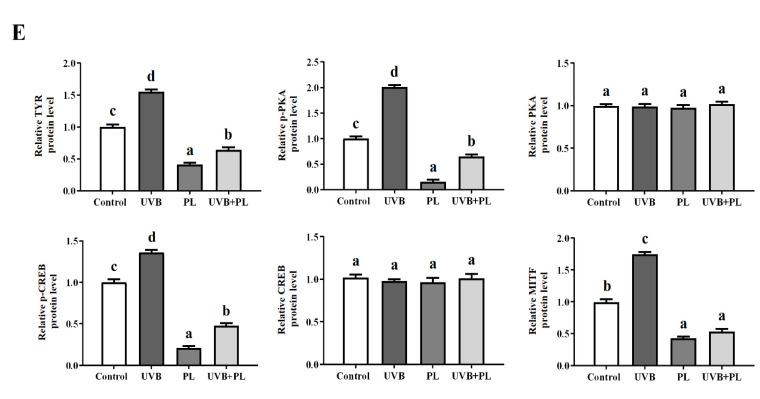
Effects of PL on melanogenesis-related parameters in B16F10 cells: (**A**) melanin content; (**B**) Tyrosinase activity; (**C**) The relative mRNA levels of TYRs; (**D**,**E**) The relative protein levels of TYR, p-PKA, PKA, p-CREB, CREB and MITF. Results are expressed as the mean ± SD (*n* = 3). Differences among the variables were assessed using Duncan’s multiple range tests. Values having different letters are significantly different (*p* < 0.05).

The effects of PL on melanogenesis-related parameters in B16F10 cells are provided in Figure 6. As shown in Figure 6A,B, compared with the control groups, UVB irradiation noticeably increased melanin content and tyrosinase activity (*p* < 0.05), and PL treatment alone noticeably decreased melanin content and tyrosinase activity in B16F10 cells (*p* < 0.05). Moreover, compared with the UVB irradiation alone groups, melanin content and tyrosinase activity in UVB+PL groups noticeably decreased in B16F10 cells (*p* < 0.05). As shown in Figure 6C, compared with the control groups, UVB irradiation noticeably increased TYR and TYRP-1 mRNA levels (*p* < 0.05), and PL treatment alone noticeably decreased TYR mRNA level (*p* < 0.05) and had no significant effect on TYRP-1 mRNA level in B16F10 cells (*p* > 0.05). Moreover, compared with the UVB irradiation alone groups, TYR and TYRP-1 mRNA levels in UVB+PL groups noticeably decreased in B16F10 cells (*p* < 0.05). There was no significant difference in TYRP-2 mRNA levels among any of the groups (*p* > 0.05).

As shown in Figure 6D,E, compared with the control groups, UVB irradiation noticeably increased TYR, p-PKA, p-CREB and MITF protein levels, and PL treatment alone remarkably or noticeably decreased TYR, p-PKA, p-CREB and MITF protein levels in B16F10 cells (*p* < 0.05). Moreover, compared with the UVB irradiation alone groups, TYR, p-PKA, p-CREB and MITF protein levels in UVB+PL groups remarkably decreased in B16F10 cells (*p* < 0.05). There were no significant differences in PKA and CREB protein levels among any of the groups (*p* > 0.05).

## 4. Discussion

Oxidative damage and photoaging are the main features of skin injury exposed to UVB for a long period of time [5,6]. In recent years, several natural extracts and artificial compounds had been used to treat UVB-induced skin oxidative damage and photoaging [7,8,9,10]. However, there were some drawbacks with the above ingredients, with them being expensive or unsafe. Sotiropoulou et al. (2021) responded that biologically active ingredients in cosmetics could be expanded to include the compounds used in food industry, such as probiotics, prebiotics and so forth [49]. Indeed, Gallinee (Ilford, Knutsford, UK) has marketed a cream that contains deactivated *Lactobacillus bacteria* along with their growth supporting prebiotics ensuring production of lactic acid and skin pH optimization [50]. Moreover, it was reported that heat-killed probiotics could effectively improve skin problems while avoiding the drawbacks of being expensive or unsafe [17,18]. Therefore, in this study we investigated the effects of PL on skin oxidative damage and photoaging as well as the related mechanisms using NHDF and B16F10 cells exposed to UVB irradiation.

### 4.1. PL Prevented UVB-Induced Cytotoxicity in NHDF and B16F10 Cells

As we know, UVB exposure interrupts membrane structural integrity, further producing toxicity in cells, thereby causing rapid cell death due to organelle malfunctions [6]. In the present research, we found that PL increased UVB-induced decrease in the viability of NHDF and B16F10 cells, suggesting that PL could prevent UVB-induced cytotoxicity in those above cells. The beneficial effects of the viability by PL in UVB-exposed skin cells may be partly attributable to the reduction in DNA damage. It has been reported that UVB radiation can cause DNA damage, thereby decreasing cell viability [51]. In this study, PL reduced UVB-induced DNA damage in NHDF and B16F10 cells, supporting our hypothesis. Moreover, UV irradiation could induce DNA damage predominantly by promoting cyclobutene pyrimidine dimers (CPDs) formation, and DNA repair was involved in xeroderma pigmentosum complementation group A (XPA) [52]. Hence, we speculated that PL-reduced DNA damage in NHDF and B16F10 cells may be partly relevant to the decreased CPDs formation and the increased XPA level. Nevertheless, the exact reason still needs further investigation.

### 4.2. PL Alleviated UVB-Induced Oxidative Damage Relating to Sirt1/PGC-1α/Nrf2 Signaling Pathway in NHDF Cells

#### 4.2.1. PL Alleviated UVB-Induced Oxidative Damage through Increasing Antioxidant Ability in NHDF Cells

Oxidative damage is the most common form of UVB radiation-induced skin injury [6]. Excessive production of ROS could lead to oxidative damage, resulting in further cell apoptosis [5]. MDA and PC contents are widely used as indices for oxidative damage (lipid peroxidation and protein oxidation, respectively) [27]. The present study showed that PL reduced UVB-induced increase in ROS, MDA and PC contents, indicating that PL alleviated oxidative damage in NHDF cells. Similar results can also be found in previous studies about heat-killed *Lactobacillus salivarius* and *Lactobacillus johnsonii*, heat-killed *Lactobacillus plantarum* 200,655 and heat-killed *Lactococcus lactis* KC24 [53,54,55].

Moreover, oxidative damage could be alleviated by nonenzymatic compound (GSH) and antioxidant enzymes (including SOD, CAT, GPx, GST and GR) [28]. In our study, PL enhanced UVB-induced decreases in GSH content as well as Cu/Zn-SOD, CAT, GPx, GST and GR activities, suggesting that PL-alleviated oxidative damage may be ascribed to an increase in antioxidant ability as a result of the enhanced GSH content and antioxidant enzymes activities in NHDF cells. This observation is consistent with the research on shrimp [56], which reported that heat-killed *Clostridium butyricum* CBG01 could decrease oxidative stress through enhancing SOD activity in the serum. Besides, a study from HT-29 cells showed that surface layer proteins, thalli ingredients obtained from *Lactobacillus strains*, could protect against hydrogen-peroxide-induced oxidative stress through elevating CAT and SOD activities [57], which is also analogous to our results.

#### 4.2.2. PL Increased Antioxidant Ability Relating to Sirt1/PGC-1α/Nrf2 Signaling Pathway in NHDF Cells

Generally, antioxidant enzyme activities are closely correlated with their mRNA levels [28]. Our study showed that PL up-regulated UVB-induced decreases in Cu/Zn-SOD, GPx, GST and GR mRNA levels, indicating that PL-elevated above antioxidant enzyme activities may be a consequence of the up-regulation of antioxidant enzymes gene expressions in NHDF cells. Moreover, Nrf2 has been demonstrated to be a critical transcription factor that promotes the transcription of antioxidant enzyme genes through binding to the antioxidant response element in the promoter region of these antioxidant enzyme genes [27]. Keap1 was identified as a Nrf2-binding protein, which depresses Nrf2 translocation to the nucleus [5]. The present study indicated that PL up-regulated UVB-induced decreases in N-Nrf2 protein expression and down-regulated UVB-induced increases in Keap1 protein expression in NHDF cells. Correlation analysis showed that N-Nrf2 protein expression was positively correlated with Cu/ZnSOD, GPx, GST and GR gene expressions and negatively correlated with Keap1 protein expression, suggesting that PL-enhanced antioxidant enzyme activities may be partly related to the activation of Nrf2/Keap1 signaling in NHDF cells. 

Sirtuin 1 (Sirt1)1, a mammalian NAD + dependent histone deacetylase, is involved in diverse cellular processes such as metabolism, cellular redox balance and resistance to oxidative stress [29]. Sirt1 could regulate important transcription factors such as PGC-1α and Nrf2, which regulates the transcription of antioxidant enzymes [28]. PGC-1α, a key enzyme associated with the activation of Nrf2, is a common target deacetylation molecule regulated by Sirt1 [58]. Some existing evidence indicates that skin oxidative damage could be attenuated by activating the SIRT1/PGC-1α/Nrf2 pathway [29]. Our study showed that PL increased UVB-induced decreases in Sirt1 and PGC-1α protein expressions in NHDF cells. Correlation analysis showed that Sirt1 protein expression was positively correlated with PGC-1α and N-Nrf2 protein expression (As shown in Table S2), indicating that the increased antioxidant enzyme activities by PL may be related to Sirt1/PGC-1α/Nrf2 signaling in NHDF cells. Moreover, a study from rats showed that inactivated pseudomonas aeruginosa could protect against oxidative stress through enhancing SOD and CAT activities relating to the Nrf2 signaling pathway in the heart [59]. It was reported that exopolysaccharides produced by *Lactobacillus rhamnosus* GG could alleviate oxidative damage through increasing SOD activity relating to the Nrf2 signaling pathway in intestinal porcine epithelial cells [60]. In addition, butyric acid and carotenoids are the two best-studied metabolites in heat-killed probiotics [61]. Similarly, previous research on butyric acid and carotenoids has demonstrated that they could enhance antioxidant ability through activating Nrf2 signaling in the rat liver and human mammary cancer cells, respectively [62,63]. These above data suggested that different heat-killed probiotics and probiotics cell lysates may alleviate oxidative damage associating with an identical signaling pathway.

Interestingly, PL had no effects on the activity and mRNA level of MnSOD as well as the mRNA level of CAT in NHDF cells. Firstly, PL increased Cu/Zn-SOD (rather than MnSOD) mRNA level and did change CAT mRNA level in NHDF cells, which may be related to valine and glucose. Cell surface proteins of heat-killed probiotics could hydrolyze into amino acids, such as valine, leucine, isoleucine and so forth [64]. It was reported that oral administration of valine could enhance the plasma glucose level in rats [65]. Sala et al. (2016) found that after exposure to constant high glucose, the Cu/Zn-SOD mRNA level increased, whereas the MnSOD and CAT mRNA levels remained unchanged in human endothelial cells [66]. Those above data suggested that PL might elevate valine abundance, thereby increasing the glucose level, further up-regulating the Cu/Zn-SOD mRNA level but not changing MnSOD and CAT mRNA levels in NHDF cells, which need further investigation. However, the reason why PL did not affect MnSOD activity is unknown and worthy of study in future work. Secondly, PL increased CAT activity (rather than CAT mRNA level) in NHDF cells. It may be explained by the reason that CAT activity is regulated not only by transcription, but also by post-translational mechanism, which requires further investigation.

As discussed above, we demonstrated that PL alleviated UVB-induced oxidative damage relating to the activation of Sirt1/PGC-1α/Nrf2 signaling pathway in NHDF cells, and may be a potential antioxidant active ingredient used in sunscreen products. To our knowledge, existing sunscreen agents are broadly classified into physical (inorganic) and chemical (organic) sunscreens based on their nature and mechanism of skin protective function. Generally, chemical sunscreen products containing active organic ingredients have a higher risk of causing skin-damaging effects, such as photoirritation, photosensitization and contact dermatitis [9,10]. In comparison, pure physical sunblock agents based on inorganic UV filters are relatively mild and are often used for children and sensitive skin. Metal oxide-based agents such as TiO_2_ and ZnO mainly act as the physical sun-protective ingredients, which have been widely used in commercial sunscreens [67]. However, they create a white-tinted matte look upon application, which is attributed to a bigger particle size [68]. Nanotechnology has been employed to produce nanoparticles (NPs) of TiO_2_ and/or ZnO, which gave a transparent appearance upon application. Unfortunately, the safety of these NPs is still a concern, although safety studies carried until now were not evident regarding their penetration into systemic circulation [67]. When exposed to UV radiation, TiO_2_ and ZnO is reported to exhibit photocatalytic activity, causing the formation of potentially harmful free radicals [69]. Moreover, compared to larger particles, nanosized minerals of TiO_2_ and ZnO are more affected by UV rays. Thus, it is crucial to protect the surrounding skin cells from these free radicals by either surface coating the NPs or by including a free radical scavenger into a sunscreen formulation. Our present study showed that PL possessed antioxidant activity, and could efficiently scavenge the photo-induced ROS to balance the oxidative level. Therefore, if included in sunscreen formulations, due to its antioxidant activity, PL may scavenge free radicals generated by UV radiation and by photocatalytic activity of TiO_2_ and ZnO. The integration of PL with metal oxide agents into other mixtures of sunscreen may perform well for its applications.

### 4.3. PL Attenuated UVB-Induced Photoaging through Anti-Wrinkling and Anti-Melanogenic Effects in NHDF and B16F10 Cells

#### 4.3.1. PL Attenuated UVB-Induced Photoaging in NHDF Cells

UVB irradiation can cause ROS generation, DNA and protein damage, inflammatory responses and a turnover of various lesions in the skin, triggering skin aging, which is also known as photoaging [51]. Cellular senescence under UVB irradiation is usually characterized by a higher staining rate of SA-β-gal positive cells [70]. In this study, we used NHDF cells as the model to investigate the effect of PL on UVB-induced photoaging by determining the percentage of SA-β-gal positive cells. We found that UVB irradiation increased the percentage of SA-β-gal positive NHDF cells, indicating that UVB irradiation accelerated photoaging in NHDF cells. Meanwhile, our results showed that PL decreased UVB-induced increases in the percentage of SA-β-gal positive NHDF cells, suggesting that PL could alleviate UVB-induced photoaging in NHDF cells. A similar result was found in the study on flavonoids, one kind of metabolites in heat-killed probiotics. Song et al. (2022) reported that total flavonoids from lycium barbarum leaves could decrease SA-β-gal-positive cell staining rate, thereby protecting against UVA-induced photoaging in human dermal fibroblasts [71].

#### 4.3.2. PL Attenuated UVB-Induced Photoaging through Exerting an Anti-Wrinkling Effect Relating to (JNK, p38)/(c-Fos, c-Jun) Signaling Pathway in NHDF Cells

Wrinkle formation is one of the most common features of skin photoaging [5]. Type I collagen, which modulates the structure of skin tissue, is one of the major markers of wrinkle development [25]. The progressive decline of type I collagen synthesis contributes to skin wrinkle formation [42]. COL1A1 is a precursor of type I collagen [25]. UV radiation increases the expression of MMPs in human skin, which are zinc-containing endopeptidases responsible for degrading the extracellular matrix (ECM) proteins such as collagen, fibronectin, elastin, and proteoglycans, contributing to photoaging [42]. In our study, PL increased UVB-induced decreases in type I collagen content and COL1A1 protein expression, and decreased UVB-induced increases in MMP-1, 2, 9 mRNA levels, indicating that PL could down-regulate MMP-1, 2, 9 mRNA levels, leading to the up-regulation of COL1A1 protein expression, further enhancing type I collagen content thereby exerting an anti-wrinkling effect in NHDF cells. Moreover, UVB-induced MMPs expressions were relevant to MAPKs signaling in skin wrinkle formation [25]. The MAPKs (including ERK, JNK and P38) pathway targets the AP-1 family of transcription factors, including c-Fos and c-Jun family members, which have been known to activate MMP transcription [42]. In this study, PL decreased p-JNK, p-p38, p-c-Fos and p-c-Jun protein expressions in NHDF cells. Correlation analysis revealed that p-JNK and p-p38 protein expressions were positively correlated with p-c-Fos and p-c-Jun protein levels as well as MMP-1, MMP-2 and MMP-9 mRNA levels (As shown in Table S2). Those data suggested that the down-regulation of MMPs expressions by PL may be partly associated with the suppression of (JNK, p38)/(c-Fos, c-Jun) signaling pathway in NHDF cells. Similarly, IM et al. (2019) reported that tyndallized *Lactobacillus acidophilus* could suppress ERK, JNK and p38 signaling, further decreasing MMP-1, MMP-2 and MMP-9 levels thereby exerting the anti-wrinkle effect in human epidermal keratinocytes cells [72]. Moreover, phenolics are also one of the components of heat inactivated probiotic cell lysates [73]. A study from human keratinocytes showed that polyphenolics could up-regulate type I collagen expression through down-regulating MMPs (1, 2, 7, 9 and 12) expression via the inhibition of MAPK (ERK, JNK and P38)/AP-1 (c-Fos and c-Jun) signaling pathway [74]. This above data revealed that heat-killed probiotics and probiotics cell lysate may exert an anti-wrinkling effect relating to the analogous signaling pathway.

Interestingly, PL had no effects on MMP-3 mRNA level or the p-ERK protein level in NHDF cells. Firstly, PL down-regulated MMP-1, MMP-2 and MMP-9 (rather than MMP-3) mRNA levels. As we know, collagenase MMP-1 serves as an initiator of extracellular matrix destruction and cooperates with other MMPs in the degradation of collagen, gelatinases (MMP-2 and MMP-9) are able to digest a number of ECM components such as collagen type I and IV, and MMP-3 cannot digest collagen type I but can activate pro-MMP-1 [75]. Thus, in this study, MMP-3 is not responsible for the activation of pro-MMP-1 involved in the degradation of type I collagen by PL. However, the reason why PL did change MMP-3 mRNA is unclear, which needs further study. Secondly, PL down-regulated p-JNK and p-p38 (rather than p-ERK) protein expressions, which may be related to leptin. Nam et al. (2022) found that heat-killed *Lactiplantibacillus plantarum* could decrease the leptin mRNA level in epididymal adipose tissue of mice [76]. It was reported that the reduction in leptin level down-regulated p-JNK and p-p38 (rather than p-ERK) protein expressions in LPS-stimulated kupffer cells of rats [77]. Thus, we hypothesized that PL might decrease the leptin level, thereby down-regulating p-JNK and p-p38 (rather than p-ERK) protein levels in NHDF cells, which needs further investigation.

#### 4.3.3. PL Attenuated UVB-Induced Photoaging through Exerting an Anti-Melanogenesis Effect Relating to PKA/CREB/MITF Signaling Pathway in B16F10 Cells

Melanogenesis is the physiological process that results in the production of melanin, a pigment that contributes to skin color [24]. Melanin plays an important role in the prevention of UV-induced skin damage [46]. However, an abnormally excessive production and accumulation of melanin could cause hyperpigmentation, which is another common feature of skin photoaging [5]. In this study, PL reduced UVB-induced increases in melanin content, suggesting that PL exerted an anti-melanogenesis effect in B16F10 cells. A similar response was also reported in the previous studies on cell-free supernatant from *Lactobacillus gasseri* and *Limosilactobacillus* fermentum JNU532 [78,79]. Moreover, melanogenesis is regulated by melanogenic enzymes, such as TYR, TYRP-1 and TYRP-2 [46]. The results in our study showed that PL decreased UVB-induced increases in tyrosinase activity and expressions (gene and protein expressions) as well as TYRP-1 mRNA level, suggesting that PL could suppress TYR and TYRP-1 activity and/or expressions, thereby exerting an anti-melanogenesis effect in B16F10 cells. Surprisingly, we found that PL down-regulated TYRP-1 (rather than TYRP-2) mRNA level in B16F10 cells, which may be associated with tyrosol. Alcohols are a kind of metabolite in heat-killed probiotics [80]. It was reported that tyrosol could inhibit TYRP-1 (rather than TYRP-2) expression in B16F0 mouse melanoma cells [81]. Therefore, we speculated that the down-regulated TYRP-1 (rather than TYRP-2) mRNA level by PL might be partly associated with the increased tyrosol level in B16F10 cells, which needs further study.

Moreover, in the various melanogenesis related different pathways, cAMP is a key physiologic signaling molecule regulating pigmentation [37]. PKA is a cAMP-dependent protein kinase that can induce CREB phosphorylation, which is well known as an important index in the upregulation of MITF expression [36]. In turn, MITF critically regulates melanogenesis related genes, such as TYR, TYRP-1 and TYRP-2 [46]. Our study showed that PL down-regulated UVB-induced up-regulation of p-PKA, p-CREB and MITF protein expressions in B16F10 cells. Correlation analysis revealed that p-PKA protein expressions were positively correlated with p-CREB, MITF, and TYR protein levels as well as TYR and TYRP-1 mRNA levels, indicating that the down-regulation of TYR and TYRP-1 expressions by PL might be partly related to PKA/CREB/MITF signaling pathway in B16F10 cells. Similar results can also be found in a study from tyndallized *Lactobacillus acidophilus* in B16F10 cells [37]. Furthermore, pyruvic acid is a metabolite in heat-killed probiotics. Zhou et al. (2019) reported that pyruvic acid could inhibit melanogenesis in B16F10 cells through PI3K/AKT, GSK3β and ROS-ERK signaling pathways [82]. This above data revealed that heat-killed probiotics and probiotics cell lysate may exert an anti-melanogenesis effect relating to the diverse signaling pathway. Even so, previous studies on B16F10 cells treated by betaine and protocatechuic aldehyde also found that PKA/CREB/MITF signaling was the pathway that suppressed melanogenesis, suggesting that this above signaling pathway plays an important role in regulating melanogenesis [83,84].

As described above, PL could attenuate UVB-induced photoaging through anti-wrinkling and anti-melanogenic effects in NHDF and B16F10 cells, respectively. PL exerted an anti-wrinkling effect through increasing type I collagen level by decreasing MMP 1, 2, 9 expressions relating to (JNK, p38)/(c-Fos, c-Jun) signaling pathway in NHDF cells, and exerted an anti-melanogenesis effect through suppressing melanogenic enzymes activity and/or expressions relating to PKA/CREB/MITF signaling pathway in B16F10 cells. Previous studies have shown that compounds acting as MMPs and melanogenic enzymes inhibitors could be useful as anti-photoaging (anti-wrinkling and whitening) agents for the formulation of cosmetic products [85,86,87]. Therefore, PL may be used as a potential active ingredient in anti-photoaging (anti-wrinkling and whitening) cosmetics.

## 5. Conclusions

**Figure 7:**
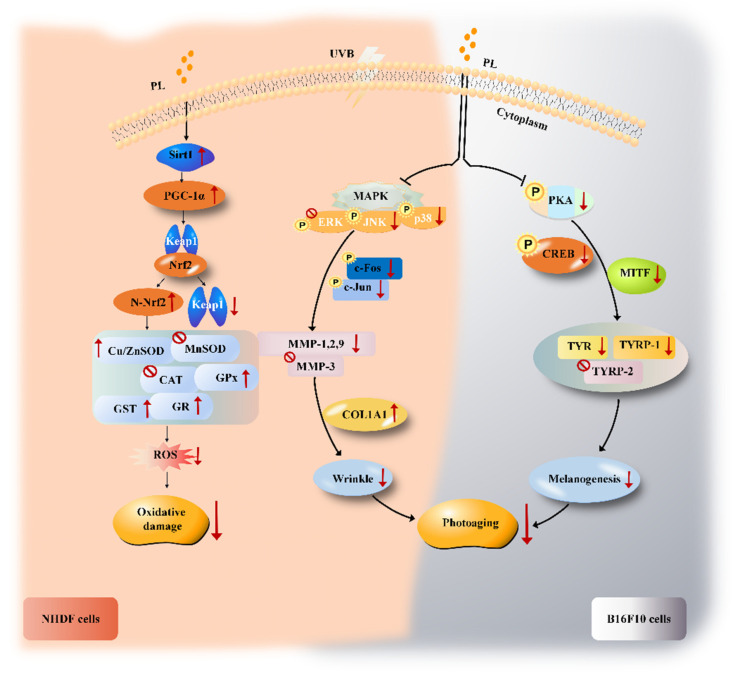
Proposed mechanism for the improvement of UVB-induced oxidative damage and photoaging by PL in NHDF and B16F10 cells. Arrows indicate positive regulation and T-bars indicate negative regulation.

In summary, the present study firstly investigated the effects of PL on UVB-induced oxidative damage and photoaging in skin cells. Firstly, PL prevented UVB-induced cytotoxicity associating with the decreased DNA damage in NHDF and B16F10 cells. Secondly, PL ameliorated UVB-induced oxidative damage through increasing GSH content as well as antioxidant enzymes activities (Cu/Zn-SOD, CAT, GPx, GST and GR) and gene expressions (Cu/Zn-SOD, GPx, GST and GR) relating to the activation of Sirt1/PGC-1α/Nrf2 signaling pathway in NHDF cells. Thirdly, PL attenuated UVB-induced photoaging noticed by decreasing the percentage of SA-β-gal positive cells in NHDF cells model. Moreover, PL could ameliorate UVB-induced photoaging through exerting anti-wrinkling and anti-melanogenesis effects in NHDF and B16F10 cells, respectively. PL down-regulated MMP-1, MMP-2 and MMP-9 expressions relating to suppress (JNK, P38)/(c-Fos, c-Jun) signaling pathway, leading to the increase in COL1A1 expression, further elevating type I collagen level, thereby exerting its anti-wrinkle effect in NHDF cells. Meanwhile, PL exerted the anti-melanogenesis effect, which was evident due to the reducing melanin content in B16F10 cells. PL reduced melanin content through decreasing tyrosinase activity and expressions (gene and protein) as well as TYRP-1 gene expression relating to the inhibition of PKA/CREB/MITF signaling pathway in B16F10 cells. The improvement of PL on oxidative damage and photoaging in NHDF and B16F10 cells are summarized in Figure 7. Our results confirm that PL may be used as a potential active ingredient for anti-oxidative damage and photoaging cosmetics. This finding provides an advanced insight into the use of probiotics as the potential antioxidant and anti-photoaging cosmetic formulation.

## Data Availability

The data presented in this study are available in this article.

## Supplementary Materials

The following supporting information can be downloaded at: https://www.mdpi.com/article/10.3390/antiox11101875/s1, Table S1: List of the primers used in the study; Table S2: List of correlation coefficient of related parameters in NHDF and B16F10 cells.
