# Supplementary material for: Heat-Killed Lacticaseibacillus paracasei Ameliorated UVB-Induced Oxidative Damage and Photoaging and Its Underlying Mechanisms
Source: Antioxidants (Basel). 2022 Sep 21;11(10):1875. doi: 10.3390/antiox11101875 (PMC9598452; doi:10.3390/antiox11101875)
Supplement: Supplementary file 1 [file antioxidants-11-01875-s001.zip › antioxidants-1889587-supplementary.pdf]

antioxidants

*Supplementary Materials*

**Table S1.** Real-time PCR primer sequences.

| Gene | Primer Sequence (Forward) | Primer Sequence (Reverse) |
|---|---|---|
| | **NHDF cells** | |
| Cu/Zn SOD | 5'-GAGTTTGGAGATAATACAGCAGGCTGT-3' | 5'- TTTCATGGACCACCAGTGTGC-3' |
| MnSOD | 5'-GTGGAGAACCCAAAGGGGAGTT-3' | 5'-GTGGAATAAGGCCTGTTGTTCCTT-3' |
| CAT | 5'-GTTACTCAGGTGCGGGCATTCTAT-3' | 5'- GAAGTTCTTGACCGCTTTCTTCTG-3' |
| GPx | 5'-CGGGACTACACCCAGATGAA -3' | 5'- TCTCTTCGTTCTTGGCGTTC-3' |
| GST | 5'-CCTGTACCAGTCCAATACCATCCT-3' | 5'-TCCTGCTGGTCCTTCCCATA-3' |
| GR | 5'-ATCCCCGGTGCCAGCTTAGG-3' | 5'- AGCAATGTAACCTGCACCAACAA-3' |
| MMP-1 | 5'-TCTCTT GGACTCTCCCATTCT-3' | 5'-AATAAGTACTGGGCTGTTCAGG-3' |
| MMP-2 | 5'-GATACCCCTTTGACGGTAAGGA -3' | 5'-CCTTCTCCCAAGGTCCATAGC-3' |
| MMP-3 | 5'-GGTGTGGAGTTCCTGATGTT-3' | 5'-TGGTCCCTGTTGTATCCTTTG-3' |
| MMP-9 | 5'-TGTACCGCTATGGTTACACTCG-3' | 5'-GGCAGGGACAGTTGCTTCT -3' |
| | **B16F10 cells** | |
| TYR | 5'-GTCCACTCACAGGGATAGCAG-3' | 5'-AGAGTCTCTGTTATGGCCGA-3' |
| TYRP-1 | 5'-ATGGAACGGGAGGACAAACC-3' | 5'-TCCTGACCTGGCCATTGAAC-3' |
| TYRP-2 | 5'-CAGTTTCCCCGAGTCTGCAT-3' | 5'-GTCTAAGGCGCCCAAGAACT-3' |
| β-actin | 5'-TGGAATCCTGTGGCATCCATGAAAC-3' | 5'-TAAAACGCAGCTCAGTAACAGTCCG-3' |

SOD: superoxide dismutase; CAT: catalase；GPx: glutathione peroxidase; GST: glutathione-S-transferase; GR: glutathione reductase; MMPs: matrix metallopeptidases; TYR, tyrosinase; TYRP: tyrosinase related protein.

**Table S2.** Correlation coefficient of related parameters in NHDF and B16F10 cells.

| Independent Parameters | Dependent Parameters | Correlation Coefficients | *p* |
|---|---|---|---|
| | **NHDF cells** | | |
| N-Nrf2 | Cu/ZnSOD | +0.853 | 0.000 |
| | GPx | +0.781 | 0.003 |
| | GST | +0.934 | 0.000 |
| | GR | +0.930 | 0.000 |
| | Keap1 | –0.931 | 0.000 |
| Sirt1 | N-Nrf2 | +0.981 | 0.000 |
| | PGC-1α | +0.998 | 0.000 |
| p-JNK | p-c-Fos | +0.932 | 0.000 |
| | p-c-Jun | +0.987 | 0.000 |
| | MMP-1 | +0.820 | 0.001 |
| | MMP-2 | +0.913 | 0.000 |
| | MMP-9 | +0.906 | 0.000 |
| p-p38 | p-c-Fos | +0.980 | 0.000 |
| | p-c-Jun | +0.992 | 0.000 |
| | MMP-1 | +0.847 | 0.001 |

| | | | |
|---|---|---|---|
| | MMP-2 | +0.979 | 0.000 |
| | MMP-9 | +0.940 | 0.000 |
| | **B16F10 cells** | | |
| p-PKA | p-CREB | +0.959 | 0.000 |
| | MITF | +0.979 | 0.000 |
| | TYR（mRNA） | +0.910 | 0.000 |
| | TYR（Protein） | +0.991 | 0.000 |
| | TYRP-1 | +0.908 | 0.000 |
